# Dosimetric parameters predicting contralateral liver hypertrophy after unilobar radioembolization of hepatocellular carcinoma

**DOI:** 10.1007/s00259-017-3845-7

**Published:** 2017-11-25

**Authors:** Xavier Palard, Julien Edeline, Yan Rolland, Samuel Le Sourd, Marc Pracht, Sophie Laffont, Laurence Lenoir, Karim Boudjema, Thomas Ugen, Vanessa Brun, Habiba Mesbah, Laure-Anne Haumont, Pascal Loyer, Etienne Garin

**Affiliations:** 1 Department of Nuclear Medicine, Cancer Institute Eugène Marquis, Avenue de la Bataille Flandres-Dunkerque, CS 44229, 35042 Rennes cedex, France; 2 University of Rennes 1, 35033 Rennes, France; 3 INSERM, INRA, Univ Rennes 1, Univ Bretagne Loire, Nutrition Metabolisms and Cancer (NuMeCan), Rennes, France; 4 Department of Medical Oncology, Cancer Institute Eugène Marquis, Avenue de la Bataille Flandres-Dunkerque, CS 44229, 35042 Rennes cedex, France; 5 Department of Medical Imaging, Cancer Institute Eugène Marquis, Avenue de la Bataille Flandres-Dunkerque, CS 44229, 35042 Rennes cedex, France; 6 Department of Hepatobiliary Surgery, CHU Pontchaillou, 2 rue Henri le Guilloux, 35033 Rennes cedex, France; 7 Department of Hepatology, CHU Pontchaillou, 35033 Rennes cedex, France; 8 Department of Medical Imaging, CHU Pontchaillou, 35033 Rennes cedex, France; 9 Department of Medical Information, Cancer Institute Eugène Marquis, Avenue de la Bataille Flandres-Dunkerque, CS 44229, 35042 Rennes cedex, France

**Keywords:** Hepatocellular carcinoma, Radioembolization, Predictive dosimetry, Liver hypertrophy, ^99m^Tc-labeled albumin, ^90^Y–loaded glass microspheres

## Abstract

**Purpose:**

This study aimed at identifying prior therapy dosimetric parameters using ^99m^Tc-labeled macro-aggregates of albumin (MAA) that are associated with contralateral hepatic hypertrophy occurring after unilobar radioembolization of hepatocellular carcinoma (HCC) performed with ^90^Y–loaded glass microspheres.

**Methods:**

The dosimetry data of 73 HCC patients were collected prior to the treatment with ^90^Y–loaded microspheres for unilateral disease. The injected liver dose (ILD), the tumor dose (TD) and healthy injected liver dose (HILD) were calculated based on MAA quantification. Following treatment, the maximal hypertrophy (MHT) of an untreated lobe was calculated.

**Results:**

Mean MHT was 35.4 ± 40.4%. When using continuous variables, the MHT was not correlated with any tested variable, i.e., injected activity, ILD, HILD or TD except with a percentage of future remnant liver (FRL) following the ^90^Y–microspheres injection  (*r* = −0.56). MHT ≥ 10% was significantly more frequent for patients with HILD ≥ 88 Gy, (52% of the cases), i.e., in 92.2% versus 65.7% for HILD < 88 Gy (*p* = 0.032). MHT ≥ 10% was also significantly more frequent for patients with a TD ≥ 205 Gy and a tumor volume (VT) ≥ 100 cm^3^ in patients with initial FRL < 50%. MHT ≥10% was seen in 83.9% for patients with either an HILD ≥ 88 Gy or a TD ≥ 205 Gy for tumors larger than 100cm^3^ (85% of the cases), versus only 54.5% (*p* = 0.0265) for patients with none of those parameters. MHT ≥10% was also associated with FRL and the Child-Pugh score. Using multivariate analysis, the Child-Pugh score (*p* < 0.0001), FRL (*p* = 0.0023) and HILD (*p* = 0.0029) were still significantly associated with MHT ≥10%.

**Conclusion:**

This study demonstrates for the first time that HILD is significantly associated with liver hypertrophy. There is also an impact of high tumor doses in large lesions in one subgroup of patients. Larger prospective studies evaluating the MAA dosimetric parameters have to be conducted to confirm these promising results.

## Introduction

Radioembolization (RE) using ^90^Y–loaded microspheres is increasingly used in a palliative setting for primary [1, 2] and secondary liver diseases [3]. The interest of RE for downstaging has also been described [2, 4, 5] especially in a recent randomized phase 2 study highlighting the superiority of RE in comparison with chemoembolization [5] opening new perspectives of surgical ablation for patients with large tumors.

Several retrospective studies have recently shown the capacity of RE to induce, after unilateral treatment, a significant hypertrophy of the opposite lobe [6–13], which is of particular interest in a neoadjuvant setting. Indeed, it is recognized that for patients with underlying liver disease, the hepatic reserve or proportion of the future remnant liver (FRL) following RE should be higher than 40 to 50% of the initial liver mass to avoid post hepatectomy liver failure [12, 14].

Historically, portal vein embolization (PVE) was used to increase the FRL of patients without cirrhosis (mainly with metastatic disease). RE has the advantage of having a direct therapeutic effect on tumors and has been recently proven to be effective in cirrhotic HCC patients [7, 12, 13]. Therefore, understanding the factors inducing liver hypertrophy after RE and developing RE for hypertrophy purposes is of major interest.

The reported mean of hypertrophy after RE varies from 29 to 45% depending on the studies and on the evaluation time. It is now recognized that the RE-mediated hypertrophy takes place over several months [13, 15, 16] and that hypertrophy is still increasing between 9 and 12 months after RE [15]. In patients treated by RE, the factors related to hypertrophy are not well described, especially dosimetric factors have not been evaluated to date except the injected activity [10] or the mean treated liver dose [12, 13, 16] without any statistically significant link identified.

Due to a minimally embolic effect in comparison with PVE, radio-induced damage and cell death within the treated liver lobe are likely to be the main inducer of the hypertrophy in the contralateral RL after RE. Thus, we hypothesized that RL hypertrophy could be at least in part in relation to the levels of injuries and induction of cell death in the injected healthy liver and/or of the tumor itself possibly in relation to the production of growth factors and cytokines [17–20]. It is mandatory to keep in mind that the threshold doses inducing damages are different in tumor versus healthy liver.

In this context, two dosimetric parameters are particularly interesting to evaluate the injected healthy liver dose (HILD) and the tumor dose especially for large lesions as recent studies have demonstrated the accuracy of MAA-based dosimetry in the prediction of tumor response [21–23] and toxicity [24, 25] for HCC patients.

The goal of this study is to evaluate the ^99m^Tc-labeled MAA dosimetric parameters prior HCC setective internal radiotherapy that would be associated with elevated contralateral hypertrophy after RE with the objective in the future to define specific dosimetric endpoints predicting liver hypertrophy.

## Material and methods

### Population

This retrospective study includes 73 consecutive unilobar HCC patients (including 94% of cases with cirrhosis) not candidates for surgery treated with ^90^Y–loaded glass microspheres (TherapSphere®, BTG UK LtD). Others selection criteria were: no whole liver injection, CT available for volume measurement performed less than 2 weeks prior to radioembolisation, MAA SCECT/CT available for dosimetric evaluation, no directed liver therapy (surgery, TACE, RFA) 3 months before RE, and no additional treatment during at least 8 weeks after RE. Written informed consent was obtained from each patient, and the use of RE was approved by the ethics committee of our institution. The indication for using RE was decided by an HCC multidisciplinary tumor board specialized in liver malignancies, including hepatobiliary surgeons. The patient’s characteristics are summarized in Table 1.

**Table 1:** Demographic and baseline characteristics of the patients (*n* = 73)

Entry (Clinical Variables)									
Gender Male/Female	Age (years)	Underlying liver disease (n)	Child classification (n)	Tumor distribution (n)	Tumor volume (cm^3^)	PVT (*n* = 31)	αFP level (kUI/l) (mean ± SD)	Platelet count (g/L)	Therapy prior RE
Male = 66	67.9 ± 8.4	Alcohol (31)	A5 (58)	Unifocal (44)	198.2 ± 177.4	Main (9)	2669 ± 9529	173 ± 81	No (65.7%)
Female = 7		HCV (7)	A6 (14)	Multifocal (29)		Branch + segmental (22)			Yes (34.3%))
		VBH (3)	B7 (1)						Surgery (4)
		Hemochromatosis (7)							Chemoembol. (16)
		NASH (11)							Sorafenib (5)
		NASH + Alcohol (10)							Radiofrequency (2)
		Non-cirrhotic (4)							

### Planning and administration of ^90^Y–loaded glass microspheres

^90^Y–loaded glass microspheres injection was classically preceded by a treatment simulation consisting of a diagnostic angiography and a liver perfusion scan (with planar and SPECT/CT acquisition) following injection of 185 MBq of ^99m^Tc-labeled MAA into the hepatic artery.

A quantitative MAA uptake analysis using a full SPECT/CT segmentation of tumor and non-tumor liver tissue was performed as previously described allowing the calculation of the injected liver dose (ILD), tumor dose (TD) and healthy injected liver dose (HILD) [22].

Glass microspheres were usually injected first week after calibration, on day 3, and 1 week after evaluating the dosimetric parameters with ^99m^Tc-labeled MAA. Fifty-two patients received a standard radiation dose of 80–150 Gy to the lobe, and 21 received a treatment intensification either at the lobe level (*n* = 18) as previously described [25] or at the segment level (*n* = 3). For all intensified patients the mean whole liver dose was always <150 Gy.

### Volumes measurement

Volume measurements of the whole liver, the injected liver (IL) and the non-injected liver corresponding to the future remnants liver (FLR), were performed before RE, at least between 4 and 8 weeks after RE and then every 12–16 weeks. Volume evaluations were interrupted at the time of progression or surgery. Three periods of volume evaluation were recorded: early (before 2 months), intermediate (between 3 and 5 months) and late (6 months and later). Volumetric assessments were carried out by the Volume Analysis software on a Syngo data-processing console display unit (Siemens) and performed on 5-mm axial slices in the portal phase. The classification of hepatic segments followed the Couinaud segmentation. The delineation was guided by anatomical landmarks and MAA SPECT/CT.

The percentage of hypertrophy (HT%) is defined as:

$$ \mathrm{HT}\%=\frac{\mathrm{FLR}\kern0.5em \mathrm{Volume}\kern0.5em \mathrm{post}\kern0.5em \mathrm{RE}-\mathrm{FLR}\kern0.5em \mathrm{Volume}\kern0.5em \mathrm{previous}\kern0.5em \mathrm{RE}}{\mathrm{FLR}\kern0.5em \mathrm{Volume}\kern0.5em \mathrm{previous}\kern0.5em \mathrm{RE}}\times 100 $$

The percentage of atrophy (AT%) is defined as:

$$ \mathrm{AT}\%=\frac{\mathrm{IL}\kern0.5em \mathrm{Volume}\kern0.5em \mathrm{previous}\kern0.5em \mathrm{RE}\hbox{-} \mathrm{IL}\kern0.5em \mathrm{Volume}\kern0.5em \mathrm{after}\kern0.5em \mathrm{RE}}{\mathrm{IL}\kern0.5em \mathrm{Volume}\kern0.5em \mathrm{previous}\kern0.5em \mathrm{RE}}\times 100 $$

Maximal hypertrophy (MHT), maximal atrophy (MAT) and their time of occurrence were recorded for the evaluation.

### Outcomes

Response of treated tumors was assessed using the European Association for the Study of the Liver (EASL) criteria at 3 months. Time to progression (TTP), response rate and disease control rate at 3 and 6 months were recorded.

Toxicities were scored using the common terminology criteria for adverse events (CTCAE) (V4). Serious liver toxicities were defined as permanent and clinically-relevant Grade ≥ III liver toxicities, manifesting within 6 months of RE.

### Statistical analysis

Quantitative values were expressed as mean ± standard deviation (SD). The volumes changes were analyzed using the Wilcoxon paired t test while the MHT was analyzed as a continuous variable and as a dichotomized one, < 10% or ≥10%, as previously described [12]. HT and AT, at the different times of analysis were compared using a paired t-test. Correlation analysis was performed using the Pearson’s test. The cut of value of HILD best predicting a MHT ≥ 10%, was defined using ROC analysis.

To evaluate the potential effect of tumor irradiation on hypertrophy, tumors were classified according to their absorbed dose and volume. The threshold tumor dose of 205 Gy described previously was used [22, 23] and the threshold volume was set as 100 cm^3^. We hypothesized that a TD ≥ 205 Gy for lesions ≥ 100 cm^3^ (TD_≥ 205Gy for TV ≥ 100cm_^3^) might be predictive of high contralateral hypertrophy levels and that TD <205 Gy whatever the tumor size, or a small tumor size (<100cm^3^) whatever the TD, (TD _< 205Gy_ or TV < 100 cm^3^), was not predictive of hypertrophy.

Factors associated with a MHT ≥ 10%, were analyzed at univariate analysis using a Chi-squared test and multivariate analysis (for independent significant variables identified at univariate analysis) using a logistic regression test. Finally, factors associated with liver toxicity were analyzed at univariate analysis using a Chi-squared test.

Median TTP was estimated using the Kaplan-Meier method.

SAS software (Version 9.3) was used for the statistical analyses with a significance threshold set at *p* ≤ 0.05.

## Results

### Variations in liver’s volumes evidencing contralateral hypertrophy

The treatment parameters (injected activity, absorbed doses) and outcomes (response rate, permanent G3 liver toxicity, secondary surgery) are presented in the Table 2.

**Table 2:** Treatment parameters and outcomes

Variables	Values
Injected Activity	2.7 ± 1.2 GBq
Injected liver dose (ILD)	149.9 ± 44.4 Gy
Tumor dose (TD)	304.5 ± 96.9 Gy
Healthy injected liver dose (HILD)	90.9 ± 31.2 Gy
Response Rate	94.5%
Secondary surgery	9 cases (12.3%)
Liver Toxicity*	4 cases (5.8%)

*Permanent and clinically-relevant Grade ≥ III liver toxicities

Livers volumes significantly changed before and after RE, at time of maximal hypertrophy of the untreated liver.

Evolution of liver volumes are presented in the Table 3.

**Table 3:** Mean liver volume (cc) and mean evolutions (cc)

	Prior injection	At time of MHT	Change	*P* value
Injected volume	899.6 ± 311.8	540.2 ± 257.7	Decrease of 359. ± 236.2	<0.0001
FRL	868.6 ± 354.1	1113.4 ± 425,3	Increase of 244.6 ± 208.9	<0.0001
Whole liver	1748.2 ± 424.2	1662.1 ± 444.4	Decrease of 87.6 ± 274.6	0.0084
FRL (%)	49.7 ± 15.9	66.7 ± 15.6	Increase of 17.0 ± 11.7	<0.0001

Fifty-eight patients (79.5%) had a MHT ≥ 10% and five patients (20.5%) had no or minimal hypertrophy, (i.e., MHT < 10%). The MHT mean was 35.4 ± 40.4% and was observed 5.9 ± 3.4 months after RE confirming the compensatory hypertrophy in the contralateral untreated liver.

The proportion of patients with a volume of non-injected liver corresponding to the future remnant liver <50% prior RE was 47.9% and significantly decreased to 13.6% only at time of MHT (*p* < 0.0001).

The mean of maximal atrophy reached 41.5 ± 9.8% of the initial volume and was observed 5.9 ± 3.1 months after RE. Only two patients (i.e., 2.7%) had no or minimal atrophy, i.e., atrophy < 10%.

**Fig. 1:**
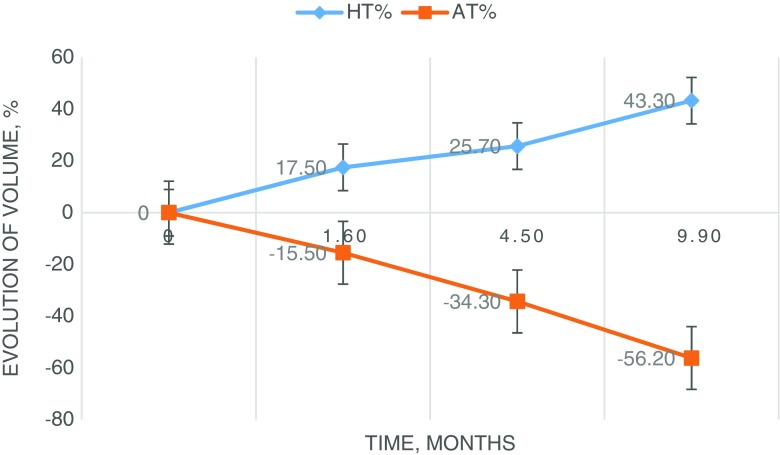
Evolution of hypertrophy (HT) of the FLR and atrophy of the treated liver (AT) regarding time

Evolution of HT and AT regarding the time of evaluation are presented in Fig. 1.

Mean time evaluation was 1.6 ± 0.4, 4.5 ± 1.1 and 9.9 ± 2.6 months for respectively early (T1), intermediate (T2) and late (T3) time points.

Mean HT significantly increased from 17.5 ± 13.8% to 25.7 ± 28.3% between T1 and T2, *p* = 0.0020 and also significantly increased to 43.3 ± 56.6% between T2 and T3, *p* = 0.0047.

Mean AT significantly increased from 15.5 ± 14.1% to 34.3 ± 15.9% between T1 and T2, *p* < 0.0001 and also significantly increased to 56.2 ± 15.8% between T2 and T3, *p* < 0.0001.

### Analysis for continuous variables

MHT mean was correlated only with the initial HR corresponding to the volume of the liver that was not injected during RE (*r* = −0.34, *p* = 0.0031) while the MAT was not significantly correlated with any parameters tested (Table 4).

**Table 4:** Correlation coefficients (r) and their *p* value for MHT% and MAT% regarding tested variables

Variables	MHT		MAT	
	r	*p* value	r	*p* value
Injected activity (GBq)	0.18	ns	0.11	ns
ILD (Gy)	0.09	ns	0.05	ns
TD (Gy)	0.07	ns	0.05	ns
TV (cc)	0.01	ns	0.07	ns
HILD (Gy)	0.10	ns	0.20	ns
HR (%)	−0.34	0.0031	0.01	ns
Platelet count (G/l)	0.04	ns	0.10	ns
MAT (%)	0.22	ns		ns

*MHT*, maximal hypertrophy; *MAT*, maximal atrophy

MHT mean was also found significantly higher for Child A patients, HR < 50% and patients who received RE at least as a second line treatment. No statistical difference was observed regarding the PVT status, hypersplenism defined as a platelet count below 100,000 G/l, (but a trend was present), response rate, toxicity, HILD threshold identified with ROC analysis and TD (TD _≥ 205Gy for TV ≥ 100cm_^3^ vs TD _< 205Gy_ or TV < 100 cm^3^) (Table 5).

**Table 5:** Comparison of means of MHT% (continuous variable) according to tested variables

Variables	Means MHT (%)	*P* value
Child A5 vs Child A6 + B7	40.1 ± 42.5 vs 17.4 ± 23.8	0.0017
HR < 50% vs ≥ 50%	48.1 ± 52.0 vs 23.8 ± 19.8	0.0092
First line vs ≥ second line	26.83 ± 23.7 vs 51.9 ± 57.8	0.0085
Hyperslenism present vs absent	19.5 ± 18.7 vs 39.2 ± 43.2	ns, but trends (*p* = 0.06)
PVT present vs absent	39.5 ± 52.4 vs 32.1 ± 27.1	ns
Response vs no response	36.4 ± 40.8 vs 12.4 ± 17.1	ns
Liver toxicity vs no liver toxicity	29.8 ± 33.2 vs 35.7 ± 40.9	ns
HILD <88Gy vs ≥ 88 Gy	33.3 ± 24.9 vs 37.7 ± 52.6	ns
HILD ≥88Gy or TD_≥205Gy for TV ≥ 100cm_^3^ vs HILD <88Gy and TD_<205Gy_ or TV < 100 cm^3^	37.4 ± 41.7 vs 24.3 ± 30.3	ns
TD _≥205Gy for TV ≥ 100cm_^3^ vs TD _<205Gy_ or TV < 100 cm^3^	40.7 ± 47.5 vs 27.4 ± 24.6	ns

### Analysis for dichotomized variables

**Fig. 2:**
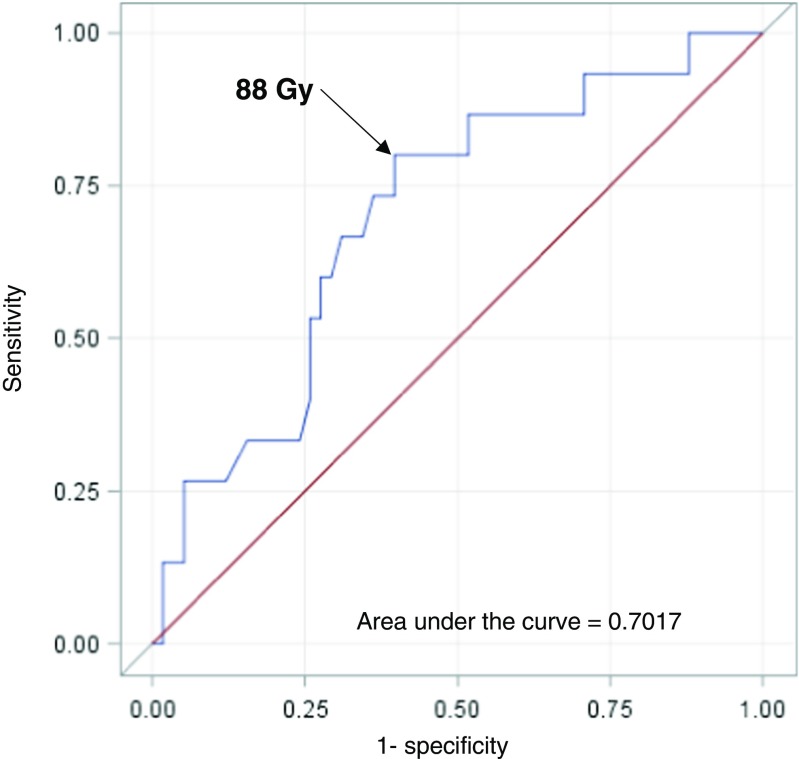
ROC curves analysis for the identification of the HIL threshold dose predicting a MHT ≥ 10%

The HILD threshold best predicting a MHT ≥10% identified at ROC analysis was 88Gy with a sensitivity of 80.9% and a specificity of 60.3% (area under the curve 0.7017) Fig. 2.

**Fig. 3:**
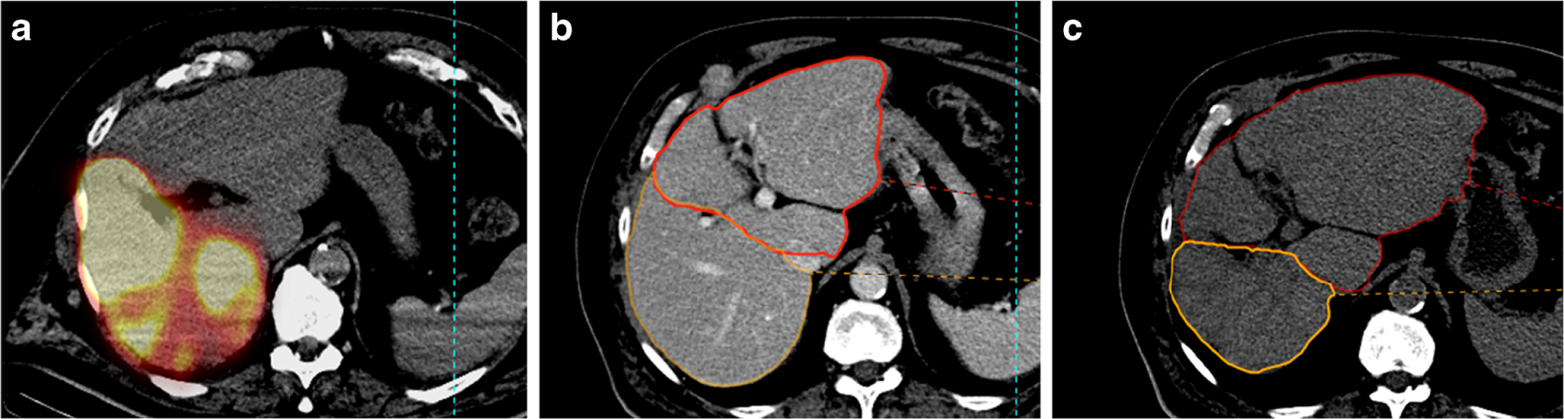
Example of hypertrophy related to a high HILD. This 74-year-old man with a Child A5 cirrhosis was treated with 2.2 GBq of ^90^Y–loaded glass microspheres injected in the RHA for HCC recurrences in the right lobe after chemoembolization. ILD was 121 Gy, HILD 114 Gy, and TD 346 Gy for a tumoral volume of 43cm^3^. The MAA scintigraphy showed an absence of perfusion of the segment 1 (**a**). The initial hepatic reserve was 52% (**b**). At 9 months, a hypertrophy of 66% of the left lobe (+ segment 1) was found and a 54% atrophy of the treated right lobe (excluding segment 1) was observed (C). The hepatic reserve after RE was 79.9%

Using univariate analysis, the MHT ≥10% was significantly associated with HILD (< or ≥88 Gy) example see Fig. 3, HR (< or ≥50%) and Child-Pugh score (A5 versus A6 + B7), the combined variable HILD ≥ 88 Gy and/or TD_≥ 205Gy for TV ≥ 100cm_^3^ (present in 85% of the cases) but not with PVT status, hypersplenism, response ratio, toxicity, treatment line, and TD (TD_≥ 205Gy and TV ≥ 100cm_^3^ vs TD _< 205Gy_ or TV_< 100cm_^3^) (Table 6). Moreover, with a multivariate analysis, the status of HILD, HR and Child status was still significantly linked with a MHT ≥ 10% (Table 6).

**Table 6:** Univariate and multivariate analysis of factors potentially associated with MHT ≥ 10%

Variables	Frequency of MHT ≥ 10%	Univariate analysis	Multivariate analysis
FLR <50% vs > 50%	92.4 vs 68.4%	*p* = 0.0203	*p* = 0.0023
HILD <88Gy vs ≥ 88Gy	65.7% vs 92.2	*p* = 0.0081	*p* = 0.0029
TD _≥205Gy and TV ≥ 100cm_^3^ vs TD _<205Gy_ or TV < 100 cm^3^	81.8 vs 75.8%	ns	–
HILD ≥88Gy or TD _≥205Gy for TV ≥ 100cm_^3^ vs HILD <88Gy and TD _<205Gy_ or TV < 100 cm^3^	83.9 vs 54.5%	*p* = 0.0265	not tested*
Child A5 vs A6 + B7	89.6 vs 40.0%	*p* = 0.0001	*p* < 0.0001
PVT present vs absent	81.8 vs 78.9%	ns	–
Hypersplenism present vs absent	71.4 vs 80.7%	ns	–
Response vs no response	81.4 vs 33.3%	ns	–
Liver toxicity vs no liver toxicity	75.0 vs 79.7%	ns	–
First line vs ≥ second line	72.9 vs 92.9%	ns, but trends (*p* = 0.0706)	–

The mixed variable HILD ≥88Gy or TD _≥ 205Gy for TV ≥ 100cm_^3^ was not tested in multivariate analysis as this variable depends on the variables HILD ≥88Gy and TD _≥ 205Gy for TV ≥ 100cm_^3^

### Subgroup analysis according to FLR and HILD

**Fig. 4:**
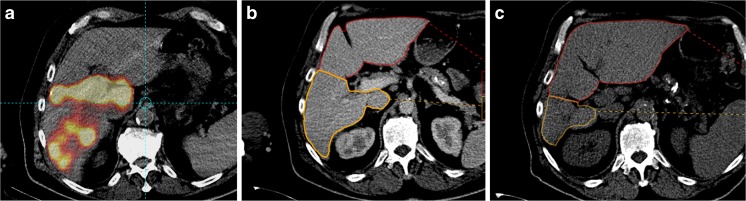
Example of hypertrophy related to a high TD for a large tumor for a patient with a very low HILD. This 66-year-old man with a Child A6 cirrhosis with a large unifocal HCC recurrence after chemoembolization was treated with 1.5 GBq of ^90^Y–loaded glass microspheres injected in the RHA. The ILD was 130 Gy, HILD was 21 Gy only while TD was 361 Gy for a tumoral volume of 151 CC. The MAA scintigraphy confirmed a perfusion of segment 1 (**a**). The initial hepatic reserve was 39% (**b**). At 6.5 months after RE, we observed a hypertrophy of 82% of the left lobe and a 52% atrophy of the right treated lobe. The hepatic reserve was then 71% (C)

FLR < 50% was seen in 47.9% of the cases at baseline. For patients with a FLR < 50%, MHT mean was significantly higher for TD _≥ 205Gy_ for TV_≥ 100cm_^3^ compared to the cases with a TD_< 205Gy_ or TV_< 100cm_^3^, with 62.3 ± 62.3% versus only 29.1 ± 25.6%, respectively (*p* = 0.0329), for example, see Fig. 4. In this group of patients, the HTM was not significantly higher for patients with HILD ≥ 88 Gy than for cases with HILD < 88 Gy with means of 52.5 ± 64.4% versus 42.2 ± 29.7%, respectively. For patients with a FLR ≥ 50%, MHT was significantly higher for cases with HILD ≥ 88 Gy than for cases with HILD < 88Gy with means of 27.6 ± 19.9% versus 17.9 ± 18.8% (*p* = 0.0454), respectively. In this group of patients, the HTM was not significantly higher for TD _≥ 205Gy_ for TV_≥ 100cm_^3^ compared to that of patients with a TD_< 205Gy_ or a TV_< 100cm_^3^ with means of 25.7 ± 24.3% versus 24.6 ± 17.6%, respectively.

HILD <88 Gy was seen in 47.9% of the cases. In this group of patients, there was a trend towards a MHT mean higher for patients with TD _≥ 205Gy_ for TV_≥ 100cm_^3^_,_ i.e., 43.9 ± 59.6% in comparison with patients with TD _< 205Gy_ or TV_< 100cm_^3^, i.e., 24.1 ± 59.6%, but it was not statistically significant. Indeed, the MHT ≥ 10% was seen for 70.8% of the patients with a TD _≥ 205Gy_ for TV_≥ 100cm_^3^ compared to a mean of only 54.5% when TD_< 205Gy_ or TV_< 100cm_^3^ (ns).

### Response, TTP and disease control rate (DCR)

Response rate was 94.5%.

At time of analysis, 79.4% of the patients had recurrence. Median TTP was 11.0 months (CI 95%: 8.5–14.0 months).

Recurrence was in the non-treated liver for 50.7% of the treated patients and in the treated liver for 28.8% of the patients.

DCR was 98.6% 3 months after RE (only one progression) and 71.2% at 6 months, with a progression in the treated liver for only 10.9% of the patients and in the non-treated liver for 17.8% of the patients.

### Toxicity analysis

HILD mean was 91.1 ± 31.9 Gy for patients without serious liver toxicity and 89.3 ± 11.5 Gy for patients with liver toxicity (*n* = 4), without statistical significance (ns). In contrast, toxicity was significantly associated with hypersplenism (*p* = 0.0224) but not with HILD (< or ≥88Gy), FLR (< or ≥50%) and Child status (A5 vs A6 + B7).

## Discussion

In this report, we have measured the dosimetric parameters following unilobar injection of ^99m^Tc-labeled MAA in patients suffering from HCC and prior ^90^Y–loaded microspheres radiotherapy in order to define specific dosimetry endpoints associated with elevated contralateral hypertrophy in non-injected liver after RE.

The first main result of this study is the clear impact of the HILD on the occurrence of a MHT ≥ 10% identified both at univariate (*p* = 0.0081) and multivariate analysis (*p* = 0.0029). MHT ≥ 10% was observed in 92.2% of the patients having a HILD ≥ 88Gy, (representing 52% of the population), while the MHT ≥ 10% was found in only 65.7% of the patients when HILD was <88 Gy. To the best of our knowledge, this is the first demonstration that a dosimetric parameter positively correlates with hypertrophy. Indeed, previous studies have failed to find any correlation between dosimetric parameters and hypertrophy. However, in these reports, the dosimetric evaluation relied on the dose to the treated liver [12, 13], which is not the best dosimetric parameter to evaluate since it is a mean dose between TD and HILD. This has also been the case in the study previously reported by Fernandez-Ros et al. [18] who have evaluated the HILD using the partition model; however, it seems that in this study HILD was only evaluated as a continuous variable. The fact that radio-induced damage is determinist and responds to a threshold can explain the absence of a link between HILD and hypertrophy in the study published by Fernandez-Ros and colleagues [18].

One way to significantly enhance hypertrophy seems to be to deliver HILD higher than 88 Gy. However, the maximal deliverable HILD without inducing liver failure is not well described. Chiesa et al. [24] found a mean healthy liver dose (mean dose to the injected and not injected healthy liver) of 75 Gy producing a probability of G2 liver toxicity of 15%. In a previous study of 71 cases, we found no link between HILD and liver toxicity but only a significant link for HILD >100 Gy and a low hepatic reserve (<30%) [25]. In the present study, no significant link was found between liver G3 permanent toxicity and HILD (<88 versus ≥88Gy). However, the maximal HILD to deliver to induce the optimal hypertrophy in case of low initial RL should be evaluated prospectively.

For the patients with a low HILD (<88 Gy, i.e., 48% of the cases), other dosimetric parameters than HILD, such as TD for large lesions, could have an impact on hypertrophy. In this situation MHT ≥ 10% was present in 70.8% of the patients with a high dose to large tumors (TD_≥ 205Gy for TV ≥ 100cm_^3^) as against only in 54.4% for low tumor dose (<205 Gy) or small tumor volume (<100cm^3^). This result was not statistically significant, but this trend highlights the potential impact of high tumor dose for large lesions on MHT. However, in one subgroup analysis (the subgroup of patients with a HR  < 50%) the parameter TD _≥ 205Gy for TV ≥ 100cm_^3^ significantly impacted the contralateral hypertrophy. In fact, in this subgroup of patients, the MHT mean was significantly higher in cases of TD _≥ 205Gy for TV ≥ 100cm_^3^ (62.3 ± 53.3%) versus only 29.1 ± 25.6% in cases with a TD _< 205Gy_ or a TV < 100 cm^3^, (*p* = 0.0329). This group of patients is of particular importance, as it represents the population that most needed the hypertrophy to be induced by RE.

It is interesting to underline that, for the same injected liver dose the lower the HILD is, the larger usually the tumors are with high doses due to the partitioning of the activity between the healthy liver and tumor. So for patients with low HILD, it seems possible to safely increase the dose in cases of large tumors, to obtain TD ≥ 205Gy, in order to stimulate hypertrophy, without reaching too high a HILD.

Finally, MHT ≥ 10% was seen in 83.9% for patients with HILD ≥ 88 Gy or a TD_≥ 205Gy for TV ≥ 100cm_^3^, representing 85% of the population, while MHT ≥ 10% was found only in 54.5% (*p* = 0.0265) for patients with none of those parameters (*p* = 0.0265).

Thus, our results are suggesting that optimizing and increasing either the HILD or the TD for large lesions could result in producing significantly higher hypertrophy for at least 85% of the patients. This data should be taken into account when discussing RE in the neoadjuvant setting of HCC. One issue not evaluated in this study is the potential impact of increasing the number of microspheres used instead of the doses. Other studies are warranted to evaluate the potential impact of microspheres number (i.e., the embolic load) on hypertrophy after RE.

After loss of the hepatic mass, the liver/body weight ratio returns to 100% because of the ability of the liver to regenerate. This process, also called Hepatostat [26, 27], allows the adjustment of its size to the overall body metabolic requirement. Using both in vitro and in vivo rodent models, it has been shown that the unique ability of differentiated hepatocytes to exit from quiescence and to proliferate is controlled by a plethora of cytokines and growth factors. The pro-inflammatory cytokines tumor necrosis factor α (TNF*α*) and interleukin 6 (IL-6) are the early stimuli during the liver regeneration allowing the priming of hepatocytes, whereas growth factors such as HGF, TGFα and EGF regulate the onset of DNA synthesis [28–30]. Many other soluble factors such as the vascular endothelial growth factor (VEGF), platelet derived growth factor (PDGF), and Augmenter of Liver Regeneration (ALR) strongly potentiate the effects of the pro-inflammatory cytokines and growth factors to achieve a complete liver regeneration and to rescue any partial defect in the secretion of one or another factor.

While the molecular mechanisms triggering the liver regeneration after partial hepatectomy have been extensively studied in rodents, the growth factors and downstream pathways regulating proliferation of hepatic cells in humans during chronic liver diseases are not well understood. In diseased human liver, the chronic loss of mature hepatocytes is not compensated by an efficient proliferation leading to the development of cirrhosis, the alterations of the liver macrophage phenotype, the abnormal production of cytokines and the appearance of progenitor cells [27]. In this pathological context, the surgical resection or the RE of HCC induce a more or less efficient liver regeneration of the non-tumoral liver although the humoral mediators remain poorly described.

However, a production of several cytokines and growth factors including VEGF, bFGF, PDGF, IL6 and HF has been recently demonstrated after RE that may constitute possible prognostic factors for the overall survival outcome [17–20]. To date, the mechanisms that trigger this production and the cell types secreting these mediators during RE are not known.

Individualizing two targets, the healthy treated liver and the tumor itself, as done in this study, seems logical in case of RE, which is responsible of tumor necrosis and can lead to injuries in the healthy liver [31]. Then, several hypothesis concerning the biological mechanisms that trigger the liver hypertrophy can be proposed. Since RE is recognized to be minimally embolic, a portal flow redistribution from the treated liver to the RL or shear-stress that could induce hypertrophy after PVE [32] is unlikely to be the main initial event with RE, even though RE is known to produce variable signs of portal hypertension with time [8, 9].

A reduction of the functional mass of the treated liver, supposedly correlated with the HILD, can be a valuable hypothesis since a significant atrophy of the treated liver is described in all RE studies [6–13]. This is the rationale for the concept of radiation lobectomy used by several authors [7, 13, 19]. In this situation, the treated lobe atrophy has been proven to be larger than the tumoral volume, confirming an atrophy of the healthy injected liver. The significant link between a HILD ≥ 88Gy and the frequency of HTM ≥ 10% found in the present study supports this hypothesis. The good tolerance of radiation lobectomy reported in all studies [6–13] in case of insufficient FLR, in comparison with surgical lobectomy, which is an immediate ablative approach, is certainly in relation to the fact that maximal atrophy is time dependent and takes several months to occur [13, 15, 16]. Therefore, the atrophy taking place after RE is certainly compensated by the occurrence of liver regeneration in the FRL in the meantime.

A second possible mechanism is the direct antitumoral effect of RE, not seen with PVE, with the potential release of mediators in case of tumor stress and necrosis (supposed to be in relation with the TD). The amount of the released mediators certainly depends upon the tumor volume and the TD. Elsewhere, HCCs are recognized to be potentially functional and, in this situation, the destruction of HCCs can lead to a reduction of the functional mass of the liver. This hypothesis is supported by the trends and significant link in one subgroup between a TD≥205Gy for TV ≥ 100cm^3^ and the frequency of HTM ≥ 10% shown in our study.

Regarding global results of hypertrophy, the MHT mean reported herein after RE, i.e., 35.4 ± 40.4%, are in agreement with previously reported data, i.e., mean (or median) MTH ranking from 29 to 45% depending on the studies and on the evaluation time [13, 15, 16].

It is now clearly established that RE significantly reduces the amount of patients with inaccurate FLR. Indeed, it has been demonstrated in previous studies that the proportion of patients with a too small FLR significantly decreased after RE. A FLR < 50% decreased from 52.9% to 32.4%, *p* = 0.013, after RE in a previous study, *p* = 0.013 [12] and a FLR < 40% also significantly decreased from 56.6% to 29.4% after RE in the study published by Fernadez-Ros et al., *p* < 0.001 [16]. Similarly in our study, the proportion of patients with a FLR < 50% prior RE, significantly decreased from 47.9% to 13.6% only at time of MHT (*p* < 0.0001).

Thus one subsequent key question is “how long we could wait for hypertrophy occurrence after RE” as it is also recognized that in this situation hypertrophy takes more time to occur than with PVE. In our study DCR was 98.6% at 3 months underlying the absence of significant risk of progression within 3 months after RE. However, DCR reduced to 71.2% at 6 months (but with progression in the treated liver in only 10.9% of the patients and in the non-treated liver for 17.8% of the patients).

So, we can recommend to wait at least 3 months after RE to evaluate hypertrophy and to check for patients operability. At 3 months, if hypertrophy is not sufficient enough, later evaluations can be recommended, especially at 6 months, as hypertrophy can take more than 3 months to occur, but in this situation a significant amount of patients will no more be candidates for surgery due to tumor progression in the non treated liver (17.8% in our experience).

Those data are confirming the interest in RE to produce significant liver hypertrophy, even in patients with underlying cirrhosis, while producing in the same way an accurate tumor control, which is not achievable while using PVE.

The main limitations of this study are its retrospective nature and the number of patients included. Despite a relative large number of patients (i.e., 73) in comparison with previously published studies [13, 16] of post RE hypertrophy in HCC (52 and 67 HCC patients in the two largest previous cohorts) subgroup analysis power is certainly not sufficient since the number of subgroup cells was below ten for the several analysis.

Elsewhere in the evaluation of the FRL after RE, the volume of the treated liver is considered as functional as the FRL itself, which is certainly not the case. Then, a simple volumetric analysis, in this situation of RE, more than likely underestimates the real percentage of the function of the FLR. In fact, an important reduction of the liver function of the treated lobe has been recently described using hepatobiliary scintigraphy in two patients out of three treated by RE [33]. In the future, the use of hepatobiliary scintigraphy should be used to evaluate more accurately the hypertrophy post RE with a more functional approach than a volumetric approach alone.

## Conclusion

This study demonstrates for the first time that HILD (< or ≥88 Gy) is significantly associated with liver hypertrophy (MHT ≥10%) with univariate and multivariate analysis underlying the robustness of this parameter. There is also a trend of the impact of high tumor dose in large lesions (TD _≥ 205Gy for TV ≥ 100cm_^3^) on MHT with a significant impact in one subgroup analysis. Larger prospective studies evaluating MAA dosimetric parameters have to be designed to confirm those results with the aim to finally define safe specific dosimetric endpoints favoring hypertrophy occurrence.
